# Traditional Mongolian Medicine Qiqirigan-8 alleviates non-alcoholic fatty liver disease via restoring gut microbiota and metabolism

**DOI:** 10.3389/fmicb.2025.1517082

**Published:** 2025-02-27

**Authors:** Dandan Yang, Wuyunsiqin, YanNiu, Hashentuya, Tana, Anna, Mingxing Ma, Wenhui Zhao, Menggenduxi, Minjie Wang

**Affiliations:** ^1^School of Traditional Mongolian Medicine, Inner Mongolia Medical University, Hohhot, China; ^2^Key Laboratory of Quality Research and Pharmacodynamic Evaluation of Traditional Chinese Medicine and Mongolia Medicine, Inner Mongolia Medical University, Hohhot, China; ^3^School of Basic Medicine, Inner Mongolia Medical University, Hohhot, China

**Keywords:** non-alcoholic fatty liver disease, gut microbiota, metabolomics, Traditional Mongolian Medicine, Qiqirigan-8

## Abstract

**Background:**

Mongolian Medicine Qiqirigan-8 (MMQ-8) is a traditional Mongolian medicine formula used to treat fatty liver disease. However, the material basis and *in vivo* metabolic process of the therapeutic effect of MMQ-8 on non-alcoholic fatty liver disease (NAFLD) remain unclear.

**Methods:**

The chemical composition of MMQ-8 was determined using Ultra-high-performance liquid chromatography-quadrupole Exactive Mass spectrometry analysis (UHPLC-QE-MS). C57BL/6J mice were fed a choline-deficient diet for 12 weeks to induce a NAFLD model. Hematoxylin and Eosin (H&E)-staining, combined with serum biochemical indexes, was used to observe liver appearance and characterize the pathological changes and functions of the liver. HE staining and Alcian Blue-Phosphoric Acid Schiff (AB-PAS) staining of the colon, along with ZO-1 immunofluorescence expression in the colon were used to reveal the effect of MMQ-8 on the disruption of the intestinal epithelial mucosal barrier in the NAFLD. The expression of intestinal tight junction genes was analyzed by qRT-PCR to observe the protective effect of MMQ-8 against intestinal epithelial mucosal barrier disruption. Fecal metagenomics and serum non-targeted metabolomics were used to reveal the effects of MMQ-8 on the gut microbiota and metabolism in mice with NAFLD. Finally, we emphasize the interaction between gut microbiota and metabolites through Spearman correlation coefficient analysis.

**Results:**

Mongolian Medicine Qiqirigan-8 contains 17 active ingredients, which can reduce hepatic steatosis and lobular inflammation in mice with NAFLD, and have protective effects against liver injury. MMQ-8 reduced the infiltration of inflammatory cells in the colon epithelium of model mice while restoring the number of goblet cells. MMQ-8 significantly enhanced ZO-1 protein expression in the colon, as well as the mRNA expression of both ZO-1 and Occludin. Fecal metagenomics results showed that MMQ-8 reduced the *Bacillota/Bacteroidota* ratio in NAFLD mice. Increased the abundance of beneficial bacteria such as *Porphyromonadaceae*, *Prevotella*, and *Bacteroidota*. and suppressed the abundance of dysfunctional bacteria, such as *Bacillota*, *Acetatifactor*, and *Erysipelotrichaceae*. Furthermore, metabolomics studies revealed that MMQ-8 intervention significantly regulated the expression of metabolites related to glutathione metabolism, butyric acid metabolism, sphingolipid metabolism, and glycerophospholipid metabolism in NAFLD mice compared to the model group. These metabolic pathways play key roles in NAFLD. According to Spearman’s correlation coefficient analysis, up-regulation of *Porphyromonadaceae*, *Prevotella*, and *Bacteroidota* after MMQ-8 intervention was negatively correlated with LPC levels in glycerophospholipid metabolic pathways, while positively correlated with PC levels. In contrast, the relationship between *Bacillota* and *Acetatifactor*, which were down-regulated after MMQ-8 intervention, was the opposite. In addition, the up-regulation of *Porphyromonadaceae*, *Prevotella*, and *Bacteroidota* after MMQ-8 intervention was positively correlated with fumaric acid, 2-oxoglutaric acid, adenosine, and L-glutathione levels, while those down-regulated after MMQ-8 intervention were positively correlated with the levels of *Bacillota*, *Acetatifactor* were negatively correlated with all the above metabolites. Thus, glutathione metabolism, butyric acid metabolism, sphingolipid metabolism, glycerophospholipid metabolism and gut microbial ecosystem are tightly intertwined in this process.

**Conclusion:**

In summary, these findings indicate that MMQ-8 has a synergistic anti-NAFLD effect through its multi-component, multi-target, gut microbiota-modulating and multi metabolic pathway characteristics. The host’s regulation of specific gut microbiota and involvement in multiple metabolic pathways may be one of the important mechanisms by which MMQ-8 exerts its therapeutic effects on NAFLD. It is worth noting that metabolic pathways such as glutathione metabolism, butyric acid metabolism, sphingolipid metabolism, glycerophospholipid metabolism, and the gut microbiota ecosystem are closely intertwined in this process.

## 1 Introduction

Non-alcoholic fatty liver disease (NAFLD) encompasses a spectrum of liver diseases that can be broadly classified into non-progressive and progressive phenotypes namely non-alcoholic fatty liver (NAFL) and non-alcoholic steatohepatitis (NASH). It is currently the most prevalent type of liver disease worldwide (Friedman et al., 2018; Lee et al., 2023), and its prevalence is increasing at an alarming rate, currently estimated at 32.4% (Riazi et al., 2022). NASH, which is characterized by liver steatosis, liver injury, inflammation, and varying degrees of fibrosis, is more likely to lead to cardiovascular, cancer, and liver-related deaths (Powell et al., 2021). The prevalence of NASH and its associated mortality is predicted to double by 2030 (Estes et al., 2018). As a result, NAFLD has become a global health problem. Although new drugs have been developed to target NAFLD, therapeutic effects have only been realized in a minority of patients. Therefore, there is an urgent need to develop new therapeutic agents for NAFLD.

The important role of the gut microbiota has been clearly demonstrated in both preclinical NAFLD models and NAFLD patients (Schnabl and Brenner, 2014). Interdependence and crosstalk between the liver and gut may also contribute to metabolic dysregulation and inflammatory responses during NAFLD (Powell et al., 2021; Pabst et al., 2023). In addition to changes in gut microbiota abundance and diversity, the potential contributing role of the gut microbiota in extraintestinal organs is realized through various bacterial metabolites such as bile acids. These metabolites are altered and involved in the pathogenesis of NAFLD, which in turn leads to intestinal barrier dysfunction and increased intestinal permeability. This promotes the translocation of bacterial material to the liver, which may stimulate the hepatic immune system and promote the development of NAFLD (Mouries et al., 2019). The gut microbiota is increasingly recognized to be involved in the treatment of NAFLD (Sharpton et al., 2021). Therefore, the prevention and treatment of NAFLD cannot be achieved without maintaining gut microbiome homeostasis and restoring metabolic homeostasis along the gut-liver axis.

For thousands of years, Traditional Chinese medicine (TCM) formulas have been widely used in the treatment of liver diseases based on the advantages of multi-target interactions (Fang et al., 2023). As a part of Chinese medicine, Mongolian medicine has a long history of clinical application. In the theory of Mongolian medicine, circulatory disorders of the “gut-liver axis” can lead to metabolic diseases, such as fatty liver, and many Mongolian medicine preparations are particularly effective in the treatment of this disease. Mongolian medicine Qiqirigan-8 (MMQ-8) is a compound containing of *Kaempferia galanga L.* (Zingiberaceae; K. galanga rhizome; Chinese name: Shannai),*Inula helenium L.* (Compositae; I. helenium rhizome and root; Chinese name: Tumuxiang), *Dolomiaea costus (Falc.)* Kasana and A.K. Pandey (Asteraceae; D. costus root; Chinese name: Muxiang), *Rheum palmatum L.* (Polygonaceae; R. palmatumradix and rhizome; Chinese name: Dahuang), *Hippophae rhamnoides L.* (Elaeagnaceae; H. rhamnoides fruit; Chinese name: Shaji), *Piper longum L.* (Piperaceae; P. longum fruit; Chinese name: Biba), *Biancaea sappan (L.)* Tod (Leguminosae;Caesalpinia sappan heart wood; Chinese name: Sumu) and *Sus scrofa L.* (Suidae; S. scrofa processed feces; Chinese name: Heibingpian). Our previous study showed that MMQ-8 could optimize lipid metabolism, reduce hepatic steatosis, inflammation protect against liver injury (Narenmandula et al., 2022). However, its active components and mechanisms remain elusive.

In this study, NAFLD mice were induced with a choline-deficient diet and MMQ-8 intervention. The results revealed that MMQ-8 significantly attenuated hepatic steatosis and liver injury. The mechanism may be by restoring the dysregulated gut microbiota and circulating metabolites.

## 2 Materials and methods

### 2.1 Ultra-high-performance liquid chromatography-quadrupole Exactive Mass spectrometry analysis (UHPLC-QE-MS) analysis

Standard diet rats, male, 6–8 weeks old, weighing 180∼220 g [License No. SCXK (Jing) 2019-0010], were purchased from SiPeiFu (Beijing) Biotechnology Co. The rats were placed in a controlled environment with a temperature of 23 ± 1°C and a humidity of 60 ± 5%, with a 12 h light/dark cycle. They were provided with a standard diet and had access to water at will. A 7 days acclimatization period was conducted under these control conditions before the start of the experiment. At the end of the acclimatization period, they were classified as blank, 0.5, 1, 2, 3, 6, 8, 12, and 24 h using the random number table method. Six rats in each group were given the same dose of MMQ-8 drug by gavage every day. A normal dose of MMQ-8 was given on day 6 with water fasting, and blood samples were collected on day 7. An Agilent ultra-high performance liquid chromatography 1,290 UPLC system with a Waters UPLC BEH C18 column (1.7 μm 2.1 × 100 mm) was used. The column temperature was 55°C, and the injection volume was 5 μL. The flow rate was set at 0.5 mL/min. The mobile phases were 0.1% formic acid aqueous solution (A) and 0.1% formic acid acetonitrile aqueous solution (B). A multi-step linear gradient elution program of 0∼11 min, 85∼25% A; 11–12 min, 25–2% A; 12–14 min, 2–2% A; 14–14.1 min, 2–85% A; 14.1–15 min, 85–85% A; 15–16 min, 85–85% a was performed using a Q Exactive Focus mass spectrometer Combined with Xcalibur software, MS and MS/MS data were acquired using IDA acquisition. In each acquisition cycle, the mass range was 100∼1,500, and the first three of each cycle were filtered to acquire the corresponding MS/MS data further. Sheath gas flow rate: 45 Arb, auxiliary gas flow rate: 15 Arb, capillary temperature: 350°C, full ms resolution: 70,000, ms/ms resolution: 17,500, collision energy: 15/30/45 in NCE mode, spray voltage: 4.0 kV (positive) or 4.0 kV (negative). The raw mass spectrometry readings were imported using XCMS software. Various tasks, including contention time correction, peak identification, extraction, integration and alignment, were performed to improve the accuracy of the data. Substance identification of peaks containing MS/MS data was performed using a self-constructed secondary mass spectrometry database and a matching method based on the corresponding cleavage rules.

### 2.2 Preparation of drugs and dosage calculation

Mongolian Medicine Qiqirigan-8 plant ratios were 207 g for *K. galanga L*; 66 g for I. *helenium L.*; 66 g for D. *costus (Falc.) Kasana and A.K. Pandey*; 41 g for R. *palmatum L.*; 248 g for H. *rhamnoides L*.; *P. longum L.* 165 g; B. *sappan (L.)* Tod for 124 g; S. *scrofa L*. for 83 g. These plants were mixed proportionally and pulverized using a pulverizer. Moreover, filtered through a 70 mesh sieve. If it could not be filtered, we continued to pulverize and sift the mixture until all the pulverized mixture passed through the filter. Finally, it was carefully dried and stored in a cool, dry place. Clinical administration requires a dose of 3 g of MMQ-8 per dose for an adult weighing 60 kg, which implies a dose of 50 mg/kg body weight. Based on body surface area, the equivalent dose ratio for humans to mice is 12.3. Therefore, the dose of MMQ-8 given to mice is 50 mg/kg of body weight (formula: MMQ-8 50 mg/kg × 12.3 g = 0.615 g/kg). Finally, after calculating the dose for each mouse, the drug was dissolved in 2 ml of H_2_O. In this study, MMQ-8-low dose group (0.615 g/kg) and MMQ-8-high dose group (1.23 g/kg) were administered by gavage for 12 weeks, and all treatments were administered by gavage once a day at a fixed time.

### 2.3 Animal models and treatment

C57BL/6J mice, male, 4–6 weeks old, 18–20 g [License No. SCXK (Jing) 2019-0010] were purchased from SiPeiFu Co (Beijing, China). Mice were kept in a normal environment during a 12 h light/dark cycle with *ad libitum* access to food and water. All mice were randomly divided into four groups (*n* = 8): control, model, MMQ-8 low dose, and MMQ-8 high dose. Except in the Control group, which was fed a standard diet, the other groups were fed a choline-deficient diet (60% fat-supplied methionine 0.1%, choline-deficient feed, batch no. 20230612 purchased from Jiangsu Xietong Pharmaceutical Bio-engineering Co (Jiangsu, China). Where the Control group did not receive any treatment, the Model group was gavaged with H_2_O, MMQ-8 low dose (0.615 g/kg), and MMQ-8 high dose (1.23 g/kg) daily for 12 weeks. All animal experiments were approved by the Animal Protection and Use Committee of Inner Mongolia Medical University (approval number: YKD202301165).

### 2.4 Biochemical analysis

Serum alanine aminotransferase (ALT, C009-2, Nanjing Jiancheng Biological Co., Ltd., China), aspartate amino-transferase (AST, C010-2, Nanjing Jiancheng Biological Co., Ltd., China), and hepatic triglyceride (TG, A110-1, Nanjing Jiancheng Biological Co., Ltd., China) levels were assayed by using commercially available diagnostic kits according to the manufacturer’s instructions.

Histopathologic evaluation

Tissue samples from the liver and colon were paraffin-embedded, sliced to a thickness of 5 μm, and put on separate slides. The sections were stained with HE (Hematoxylin, G1004, Solarbio, China; Eosin, BA-4024, Basso, China) and ABPAS (G1285, Solarbio, China), respectively, and observed by light microscopy. HE staining of the liver was used for NAFLD activity score (NAS) (Qu et al., 2023). Steatosis was scored from 0 to 3 (0: < 5% steatosis; 1: 5–33%; 2: 34–66%; 3: > 67%). Hepatocyte ballooning was scored from 0 to 2 (0: normal hepatocytes, 1: normal-sized with pale cytoplasm, 2: pale and enlarged hepatocytes, at least 2-fold). Lobular inflammation was scored from 0 to 3 based on foci of inflammation counted at 20X (0: none, 1: < 2 foci; 2: 2–4 foci; 3: R4 foci). NAS was calculated as the sum of steatosis, hepatocyte ballooning and lobular inflammation scores.

### 2.5 Immunofluorescence staining

Colon sections were prepared according to the procedure described above, and the sections were incubated with anti-ZO-1 (21773-1-AP, proteintech, China) primary antibody overnight at 4°C, followed by incubation with fluorophore-coupled secondary antibody (5220-0336, SeraCare, China) for 1 h. Cell nuclei were stained with DAPI (C0065, Solarbio, China). Cell nuclei were stained with DAPI (C0065, Solarbio, China). Images were acquired using a fluorescence microscope (BX51, Olympus, Japan).

### 2.6 Quantitative real-time polymerase chain reaction (qRT-PCR)

RNA was extracted from mice colon tissues using TRIzol reagent (Lot#B04, ProbeGene, China) according to the manufacturer’s instructions, and RNA was reverse-transcribed into cDNA using a reverse transcription kit (FSQ-201, TOYOBO, Japan). Next, qRT-PCR was performed on a QuantStudio3 Real-Time PCR Instrument (A28132, ThermoFisher, United States) using SYBR Green real-time PCR Master Mix (QPK-201, TOYOBO, Japan). Details of the messenger RNA-specific primers for Occludin, ZO-1 are shown in Table 1. Relative mRNA levels were determined using the comparative Ct method, with GAPDH mRNA as the reference gene, and Equation 2^–ΔΔCT^.

**TABLE 1:** Primiers used in this study for quantitative real-time polymerase chain reaction (qRT-PCR).

Gene	Forward primer (5′ to 3′)	Reverse primer (5′ to 3′)
GAPDH	AGGTCGGTGTGAA CGGATTTG	TGTAGACCATGTAGTT GAGGTCA
Occludin	TTGAAAGTCCACCT CCTTACAGA	CCGGATAAAAAGA GTACGCTGG
ZO-1	GCCGCTAAGAGCA CAGCAA	TCCCCACTCTGAAAA TGAGGA

### 2.7 Fecal metagenomics

Five mice feces were taken from each of the three groups, the Control group, the Model group and the MMQ-8 group, and DNA was isolated in a sterile environment. The DNA samples that passed the test were randomly interrupted by a Covaris ultrasonic crusher to form fragments with a length of about 350 bp. and inserted into metagenome shotgun sequencing libraries. Paired-end sequencing was performed using the Illumina HiSeq high-throughput sequencing platform. The raw data were subjected to quality control and host filtering to obtain Clean Data. Then, functional annotation and abundance analysis of metabolic pathways (KEGG) were performed; the α-diversity of fecal bacteria was calculated based on the normalized OTU table of R package Vegan; based on the species abundance table and functional abundance table, abundance clustering analyses such as PCA, PcoA, and NMDS, and multivariate statistical analysis of LEfSe, as well as comparative metabolic pathway analyses, were carried out to excavate the differences in the species compositions and functional compositions among samples; and the differences in species and functional compositions among samples were explored. Functional composition differences.

### 2.8 Serum untargeted metabolomics

A 100 μL serum sample from five mice in each of the Control, Model, and MMQ-8 groups was placed in an EP tube, and 400 μL of 80% methanol in water was added to the tube. The sample was vortexed and shaken, allowed to stand for 5 min on an ice bath, and centrifuged at 15,000 *g* for 20 min at 4°C. A certain amount of supernatant was diluted with mass spectrometry-grade water to 53% methanol; the supernatant was collected by centrifugation at 15,000 *g* for 20 min at 4°C and injected into the LC-MS for analysis. The data (.raw) files were imported into CD 3.3 library software for processing. The data were pre-processed, identified with metabolites, and finally analyzed statistically. Data were transformed and subjected to principal component analysis (PCA) and partial least squares discriminant analysis (OPLS-DA) using metabolomics data processing software meta X. The criteria for differential metabolite screening were VIP > 1, *P*-value < 0.05 and FC ≥ 1.2 or FC ≤ 0.5. The KEGG database was used to study the functions and metabolic pathways of metabolites, which were considered to be enriched when x/n > y/n and significantly enriched when the *P-*value of the metabolite pathway < 0.05. Screening of KEGG pathways related to NASH was performed using the microbiology platform^1^ for histogram plotting.

### 2.9 Combined analysis of metagenomics and untargeted metabolomics

The above gut microbiota and non-targeted metabolomics data were subjected to Spearman correlation analysis using the cloud platform^2^.

### 2.10 Statistical analysis

Data are expressed as mean ± SEM. Non-parametric Kruskal Wallis one-way analysis of variance (ANOVA) was used to compare the ranking data between different groups. When the difference is significant (*P* < 0.05), then the Mann-Whitney U test is performed, which is determined by pairwise comparison of significant differences in each group.

## 3 Results

### 3.1 Identifying the potential active components of MMQ-8 through UHPLC-QE-MS

**FIGURE 1:**
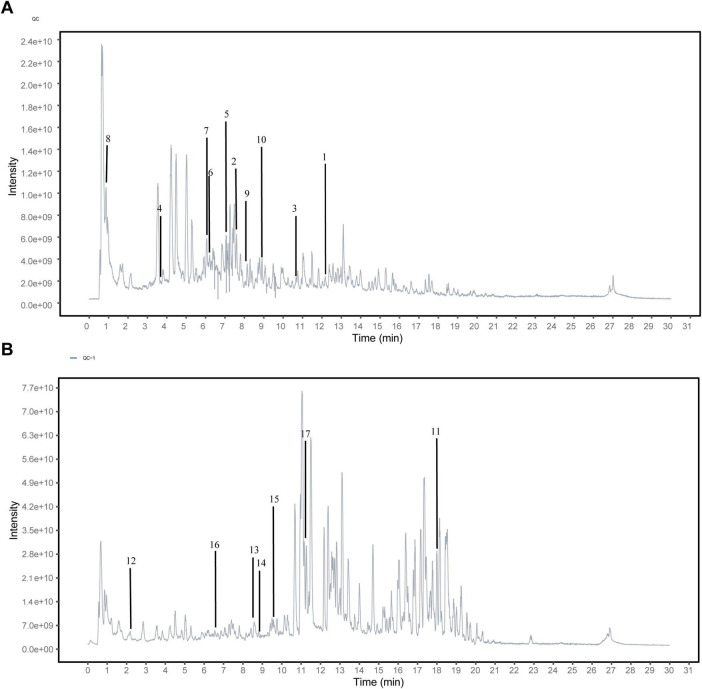
Identifying the potential active components of Mongolian Medicine Qiqirigan-8 (MMQ-8) through Ultra-high-performance liquid chromatography-quadrupole Exactive Mass spectrometry analysis (UHPLC-QE-MS). **(A)** Active components of MMQ-8 in NEG electrospray ionization modes in serum. **(B)** Activecomponents of MMQ-8 in POS electrospray ionization modes in serum.

To further clarify the effective material basis of MMQ-8 to improve NAFLD, we analyzed MMQ-8-treated serum samples and observed the number of peaks and their responses in the chromatograms (Figures 1A, B). Compounds were identified by considering retention times, molecular ions, major fragment ions, and information from published articles and online databases. Subsequently, 17 potentially active components of MMQ-8 were screened. These identified compounds mainly belonged to the flavonoid class. Table 2 gives detailed information about the identified compounds in the serum samples, including retention times, precise molecular weights, etc.

**TABLE 2:** Identifying the active components of Mongolian Medicine Qiqirigan-8 (MMQ-8) through ultra-high-performance liquid chromatography-quadrupole Exactive Mass spectrometry analysis (UHPLC-QE-MS).

Peak.No	Name	InChIKey	Formula	Class	rtmed
1	[(2R)-2-[(E,2S,4R)-4,6-dimethyloct-6-en-2-yl]-6-oxo-2,3-dihydropyran-3-yl](2E,4E,6S)-8-hydroxy-6-(hydroxymethyl)-4-methylocta-2,4-dienoate	OHRGHFXATDKGOV-MDKHPKFKSA-N	C25H38O6	Miscellaneous	743.5815
2	Afzelin	SOSLMHZOJATCCP-AEIZVZFYSA-N	C21H20O10	Flavonoids	450.6265
3	Atractylenolide III	FBMORZZOJSDNRQ-UHFFFAOYNA-N	C15H20O3	Sesquiterpenoids	636.529
4	Homoplantaginin	GCLAFEGUXXHIFT-IWLDQSELSA-N	C22H22O11	Flavonoids	224.136
5	Isopeonol	XPHIPEXPAGCEBM-UHFFFAOYSA-N	C9H10O3	Phenols	426.582
6	Kaempferol-3-O-rutinoside	RTATXGUCZHCSNG-QHWHWDPRSA-N	C27H30O15	Flavonoids	383.3135
7	Liquiritin	DEMKZLAVQYISIA-ZRWXNEIDSA-N	C21H22O9	Flavonoids	380.888
8	N-Acetyl-DL-glutamic acid	RFMMMVDNIPUKGG-UHFFFAOYSA-N	C7H11NO5	Amino acid derivatives	56.5073
9	Syringin	QJVXKWHHAMZTBY-GCPOEHJPSA-N	C17H24O9	Organic oxygen compounds	485.174
10	Trifolirhizin	VGSYCWGXBYZLLE-QEEQPWONSA-N	C22H22O10	Isoflavonoids	537.299
11	(1S,4aR,6aS,6bR,9R,10R,11R,12aR,14bS)-1,10,11-trihydroxy-9-(hydroxymethyl)-2,2,6a,6b,9,12a-hexamethyl-1,3,4,5,6,6a,7,8,8a,10,11,12,13,14b-tetradecahydropicene-4a-carboxylic acid	IFIQVSCCFRXSJV-ZIZFEDMCSA-N	C30H48O6	Terpenoids	1074.8
12	6-(1,1-DIMETHYLALLYL)-2-(1-HYDROXY-1-METHYLETHYL)-2,3-DIHYDRO-7H-FURO[3,2-G]CHROMEN-7-ONE	JCDLLLXYAICSQV-INIZCTEOSA-N	C19H22O4	Phenylpropanoids	143.189
13	7,8-Dihydroxyflavone	COCYGNDCWFKTMF-UHFFFAOYSA-N	C15H10O4	Flavonoids	513.7995
14	Alkannin	NEZONWMXZKDMKF-JTQLQIEISA-N	C16H16O5	Quinones	528.2735
15	Karakoline	HKQZUYOVMYOFIT-UHFFFAOYSA-N	C22H35NO4	Terpenoids	568.1
16	Tectoridin	CNOURESJATUGPN-UDEBZQQRSA-N	C22H22O11	Flavonoids	400.196
17	Tetrahydropiperine	APZYKUZPJCQGPP-UHFFFAOYSA-N	C17H23NO3	Alkaloids	679.394

### 3.2 MMQ-8 attenuates choline-deficient diet-induced NAFLD and hepatic injury

**FIGURE 2:**
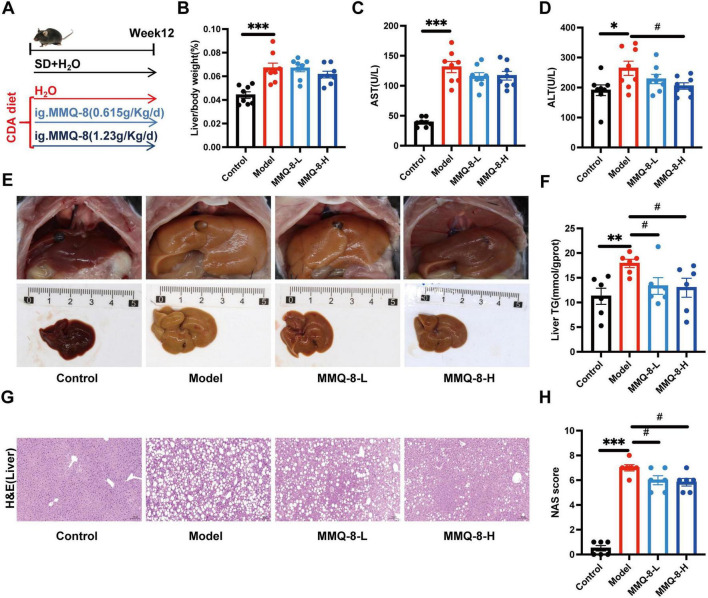
Mongolian Medicine Qiqirigan-8 (MMQ-8) attenuates choline-deficient diet-induced non-alcoholic fatty liver disease (NAFLD) and liver injury. **(A)** Schematic representation of the experimental design. C57BL/6J mice were fed a standard diet (SD) or a choline deficiency diet (CDA), and mice were randomized to receive MMQ-8 at doses of 0.615 g/kg/day, 1.23 g/kg/day, or H_2_O, respectively, for 12 weeks (*n* = 8 per group). **(B)** Liver/body weight (*n* = 8 per group). **(C,D)** Serum levels of AST and ALT (*n* = 8 per group). **(E)** Gross appearance of the peritoneal cavities (*n* = 6 per group). **(F)** Liver TG levels (*n* = 6 per group). **(G)** Representative images of mice liver HE, scale bar, 100 μm (*n* = 6 per group). **(H)** NAFLD activity score (NAS) (*n* = 6 per group).

To investigate the pharmacological effects of MMQ-8 on NAFLD and liver injury, we used a choline-deficient diet-induced NAFLD model. C57BL/6J mice were randomly assigned to Control, Model, MMQ-8 low-dose, and MMQ-8 high-dose groups for 12 weeks (Figure 2A). Choline-deficient diet feeding significantly increased liver/body weight (Figure 2B), serum AST (Figure 2C), and ALT (Figure 2D) levels leading to hepatic injury as compared to the Control group. Importantly, MMQ-8 treatment significantly reduced hepatic ALT levels in NAFLD mice, with a protective effect against liver injury. In addition, the gross appearance of the peritoneal cavities (Figure 2E) and liver TG (Figure 2F) results showed that MMQ-8 could attenuate hepatomegaly and reduce hepatic steatosis in NAFLD mice. Pathological analysis of liver HE staining showed hepatic steatosis, hepatocyte balloon-like lesions, and significant lobular inflammatory infiltration in the Model group mice compared with the Control group. In contrast, liver steatosis and lobular inflammation were significantly reduced after MMQ-8 intervention (Figure 2G), and the NAS activity score was decreased (Figure 2H), which was consistent with previous reports (Narenmandula et al., 2022). In conclusion, these results suggest that MMQ-8 can alleviate hepatic steatosis and liver injury in NAFLD mice.

### 3.3 MMQ-8 can repair intestinal epithelial mucosal barrier disruption in NAFLD mice

**FIGURE 3:**
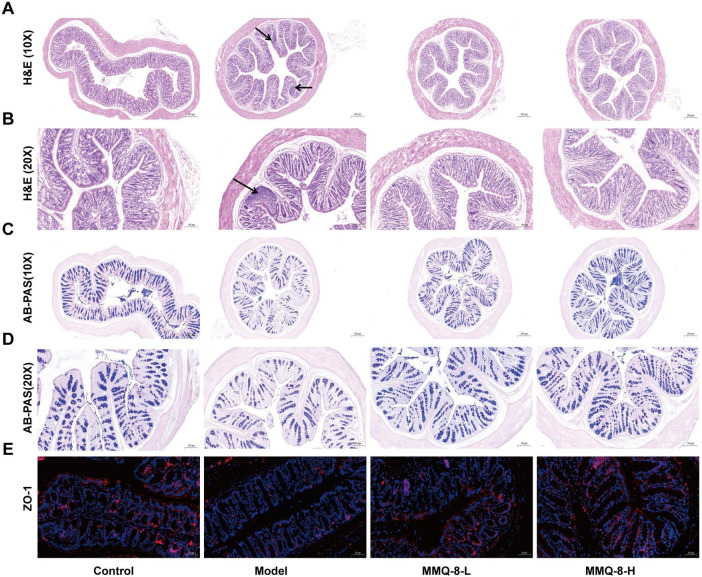
Mongolian Medicine Qiqirigan-8 (MMQ-8) can repair intestinal epithelial mucosal barrier disruption in non-alcoholic fatty liver disease (NAFLD) mice. **(A)** HE staining of mice colon, scale bar, 200 μm (*n* = 6 per group). **(B)** HE staining of mice colon, scale bar, 100 μm (*n* = 6 per group). **(C)** AB-AS staining of mice colon, scale bar, 200 μm (*n* = 6 per group). **(D)** AB-PAS staining of mice colon, 100 μm (*n* = 6 per group). **(E)** Immunofluorescence staining of ZO-1 (red) in mice colon. Nuclei were labeled with DAPI (blue), scale bar, 50 μm (*n* = 3).

In order to further study the repair effect of MMQ-8 on the intestinal epithelial mucosal barrier of NAFLD, HE staining of the colon was performed (Figures 3A, B). It was found that the colonic surface structure was complete in the model group, but inflammatory cell infiltration could be seen. However, the villi of the colonic mucosa of MMQ-8 mice were more neatly arranged than that of the model group, and the inflammatory cell infiltration was reduced. The trend of the results of AB-PAS staining (Figures 3C, D) was similar to that of HE staining. Compared with the Control group, the epithelial cells and goblet cells in the superficial layer of the colonic mucosa of mice were full and rounded, while the epithelial cells and goblet cells in the superficial layer of the colonic mucosa of the mice in the Model group were shrunken and their number appeared to be reduced to different degrees. However, MMQ-8 treatment restored the reduced number of goblet cells compared to the Model group. Immunofluorescence staining (Figure 3E) showed that the fluorescent expression of ZO-1 was reduced in the colon of mice in the Model group compared with the Control group. In contrast, the expression of ZO-1 was significantly enhanced in the colon of both MMQ-8 groups. In summary, MMQ-8 may significantly promote the repair of intestinal damage.

### 3.4 MMQ-8 significantly up-regulates the expression of Occludin ZO-1 in NAFLD mice

**FIGURE 4:**
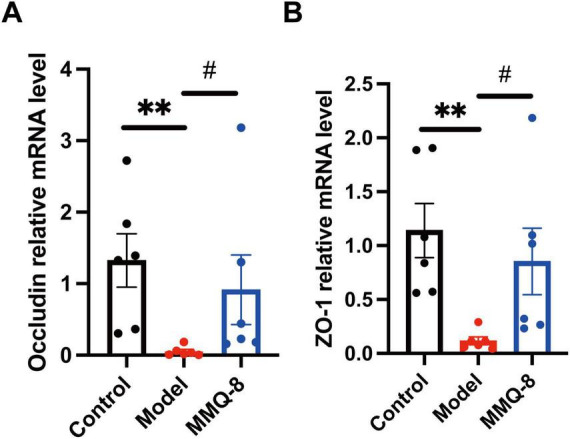
Mongolian Medicine Qiqirigan-8 (MMQ-8) up-regulates the expression of Occludin, ZO-1 in non-alcoholic fatty liver disease (NAFLD) mice. **(A)** Quantitative real-time polymerase chain reaction (qRT-PCR) was used to determine the gene Occludin mRNA levels in the colon of mice in the Control, Model and MMQ-8 groups. Gene expression was normalized to GAPDH mRNA levels. Each group *n* = 6. **(B)** qRT-PCR was used to determine the gene ZO-1 mRNA levels in the colon of Control, Model and MMQ-8 mice. Gene expression was normalized to GAPDH mRNA levels. Each group *n* = 6. **P < 0.01, compared to Control group. #*P* < 0.05, compared to the Model group.

Therefore, we further analyzed the colon-intestinal barrier tight junction-related genes by qRT-PCR. The results showed that the expression of ZO-1 (Figure 4A) and Occludin mRNA (Figure 4B) was significantly suppressed in the colon of the Model group. In contrast, MMQ-8 treatment significantly increased expression in the colon, suggesting a possible improvement in intestinal barrier tight junctions.

### 3.5 MMQ-8 significantly modulates alterations in the fecal microbiota of NAFLD mice

**FIGURE 5:**
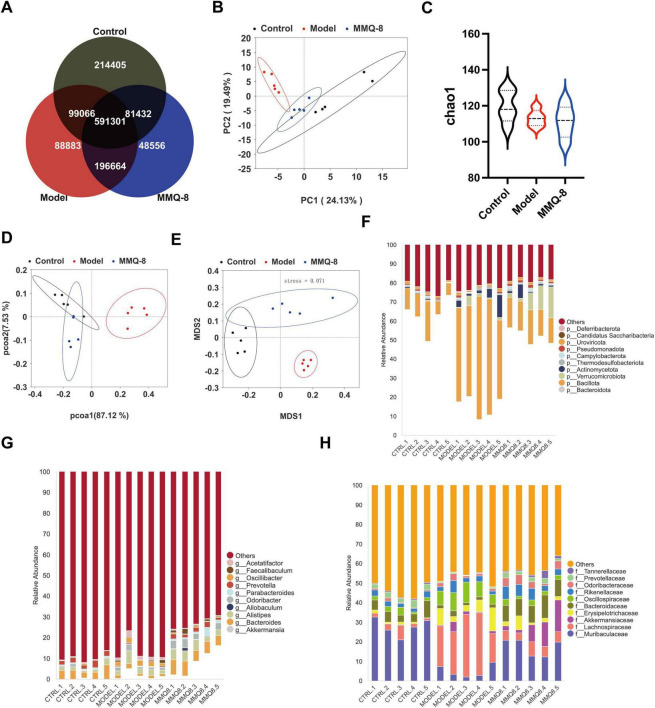
Mongolian Medicine Qiqirigan-8 (MMQ-8) significantly modulates alterations in the faecal microbiota of non-alcoholic fatty liver disease (NAFLD) mice (*n* = 5). **(A)** Shared and unique Observed Operational Taxonomic Units (OTUs) for three groups. **(B)** Principal component analysis (PCA). **(C)** α-diversity (Chao1). **(D)** PCoA analysis. **(E)** NMDS analysis. **(F)** Relative abundance of the top 10 abundant microbial species at the phylum level. **(G)** Relative abundance of the top 10 abundant microbial species at the genus level. **(H)** Relative abundance of the top 10 abundant microbial species at the family level.

To explore whether MMQ-8 improves the gut microbiota to attenuate NAFLD, we used fecal metagenomics. Firstly, the gut microbiota of the Control, Model, and MMQ-8 groups was subjected to the Veen diagram (Figure 5A), and it was found that the three groups of samples shared 591,301 species. Based on gut microbiota abundance, the results of principal component analysis (PCA) (Figure 5B) showed a clear separation of the three groups, with MMQ-8 being closer to the Control group, suggesting that the microbiota species compositions were closer to each other. In addition, the α-diversity index of the Model group was relatively low, and the α-diversity index of the intestinal microbiota (Figure 5C) was not significantly improved after MMQ-8 treatment, suggesting that MMQ-8 did not have a significant effect on the diversity of the various microbiota in the intestinal tract. PcoA (Figure 5D) and NMDs (Figure 5E) analyses were next performed to assess the β-diversity among the three groups, and there were significant differences among the three groups, suggesting that the three groups exhibited different microbiota clustering. Thus, MMQ-8 treatment-induced changes in gut microbial community structure in NAFLD mice without affecting microbiota α-diversity. Notably, MMQ-8 treatment significantly modulated microbiota abundance at the phylum level (Figure 5F), genus level (Figure 5G) and family (Figure 5H) level.

### 3.6 Specific changes in gut microbiota after MMQ-8 treatment in NAFLD mice

**FIGURE 6:**
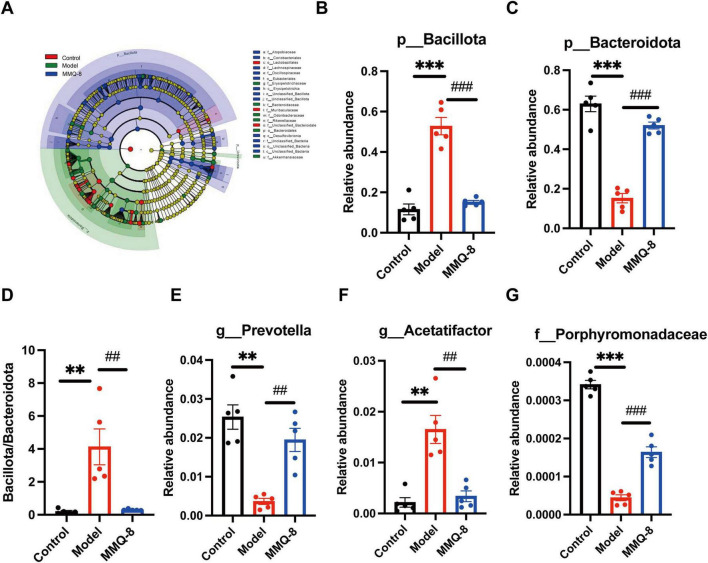
Specific changes in gut microbiota after Mongolian Medicine Qiqirigan-8 (MMQ-8) treatment in non-alcoholic fatty liver disease (NAFLD) mice. **(A)** LEfSe analysis of the gut microbiota (LDA score > 4). **(B)** Relative abundance of Bacillota at the phylum level. **(C)** Relative abundance of Bacteroidota at the phylum level. **(D)** Bacillota/Bacteroidota ratio. **(E)** Relative abundance of Prevotella at the genus level. **(F)** Relative abundance of Acetatifactor at the genus level. **(G)** Relative abundance of Porphyromonadaceae at the family level.

Next, linear discriminant analysis effect size (LDA Effect Size, LEfSe) identified several families (LDA scores > 4) that distinguished the three gut microbiota groups. Among them, *Erysipelotrichaceae* was significantly dominant in the Model group, and *Akkermansiaceae* was significantly dominant in the MMQ-8 group (Figure 6A). At the phylum level, there was a significant increase in the relative abundance of *Bacillota* (Figure 6B) and a significant decrease in the relative abundance of *Bacteroidota* (Figure 6C) in the model group compared to the control group. In contrast, MMQ-8 intervention reversed the above changes and significantly reduced the *Bacillota/Bacteroidota* ratio in NAFLD mice (Figure 6D). At the genus level, MMQ-8 significantly increased *Prevotella* (Figure 6E) abundance and significantly down-regulated *Acetatifactor* (Figure 6F) abundance. *Porphyromonadaceae* abundance was significantly up-regulated at the family level (Figure 6G).

### 3.7 MMQ-8 alters gut microbiota function and metabolic pathways in NAFLD mice

**FIGURE 7:**
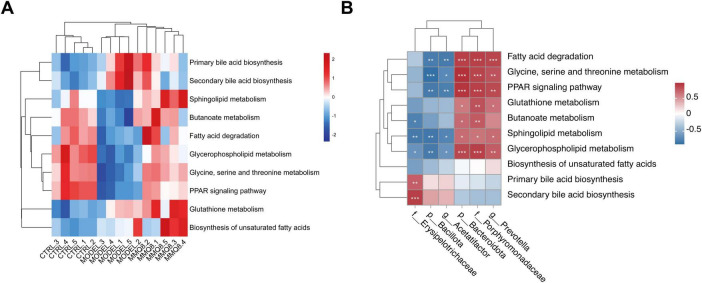
Mongolian Medicine Qiqirigan-8 (MMQ-8) alters gut microbiota function and metabolic pathways in non-alcoholic fatty liver disease (NAFLD) mice. **(A)** Heatmap of hierarchical cluster analysis of metabolic pathways among the three groups. **(B)** The predictive function of the gut microbiota between altered Spearman correlation analysis. Red indicates a positive correlation, and blue indicates a negative correlation. *P < 0.05; **P < 0.01; ***P < 0.001.

Furthermore, functional prediction of the gut microbiota showed significant changes in metabolic pathways after MMQ-8 intervention. Energy metabolism, amino acid metabolism, and lipid metabolism were changed in the Model group compared to the Control group. Significantly, MMQ-8 treatment reversed these changes. Compared with the Model group, metabolic pathways such as amino acid metabolism, bile acid metabolism, butyric acid metabolism and sphingolipid metabolism, glycerophospholipid metabolism, fatty acid degradation, and PPAR signaling pathway appeared to be significantly altered after MMQ-8 treatment (Figure 7A). Increased levels of *Porphyromonadaceae*, *Prevotella*, and *Bacteroidota* after MMQ-8 treatment were generally positively correlated with lipid metabolism, butyric acid metabolism, and amino acid metabolism and negatively correlated with the abundance of *Bacillota*, *Acetatifactor*, and *Erysipelotrichaceae* (Figure 7B). Therefore, it is hypothesized that MMQ-8 regulates the gut microbiota, thereby affecting metabolic pathways in NAFLD mice.

### 3.8 MMQ-8 ameliorates serum metabolic alterations in NAFLD mice

**FIGURE 8:**
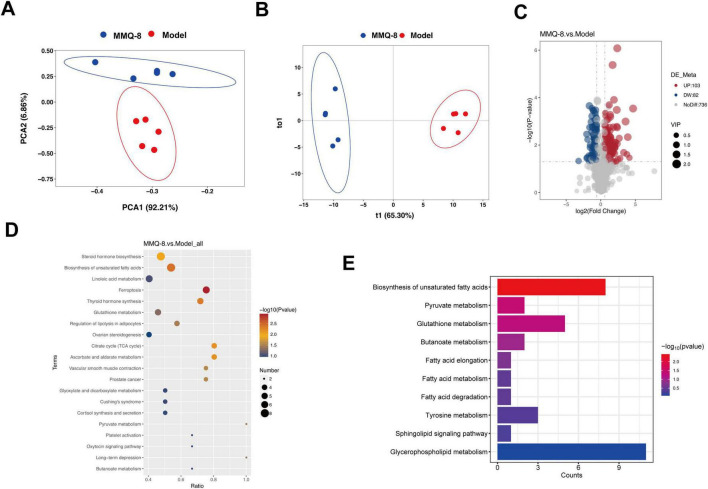
Mongolian Medicine Qiqirigan-8 (MMQ-8) improves serum metabolic alterations in non-alcoholic fatty liver disease (NAFLD) mice. **(A)** Principal Component Analysis (PCA). **(B)** Orthogonal Partial Least Squares Discriminant Analysis (OPLS-DA). **(C)** Scatter plots of differential metabolites between MMQ-8 and Model groups. Red dots represent significantly up-regulated metabolites (*p* < 0.05 and FC > 1.2), and green dots represent down-regulated metabolites (*p* < 0.05 and FC < 0.5). **(D)** Kyoto Encyclopedia of Genes and Genomes (KEGG) enrichment analysis between MMQ-8 and Model groups. **(E)** KEGG enrichment analysis associated with non-alcoholic steatohepatitis (NASH).

To further explore the metabolic mechanisms on which MMQ-8 ameliorates NASH, we performed serum untargeted metabolomics. Principal component analysis (PCA, Figure 8A) and orthogonal partial least squares discriminant analysis (OPLS-DA, Figure 8B) showed that different metabolic changes were found in the serum of MMQ-8 and Model group mice. Scatterplot visualization of the two groups of differentially expressed metabolites using *P*-value, and Fold Change (Figure 8C) revealed that MMQ-8 significantly up-regulated 103 metabolites and down-regulated 82 metabolites compared with the Model group. We then performed a KEGG enrichment analysis of these differential metabolites and found that unsaturated fatty acid synthesis was the most significant (Figures 8D, E).

### 3.9 MMQ-8 alters serum-specific metabolic pathways in NAFLD mice

**FIGURE 9:**
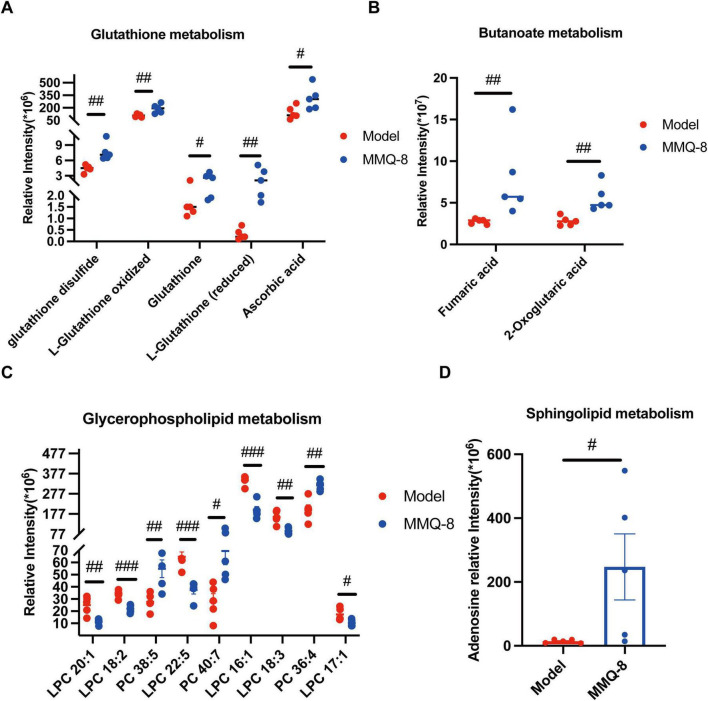
Mongolian Medicine Qiqirigan-8 (MMQ-8) Alters Serum-Specific Metabolic Pathways in non-alcoholic fatty liver disease (NAFLD) mice. **(A)** Relative expression of metabolites associated with the glutathione metabolic pathway. **(B)** Relative expression of metabolites related to the butyric acid metabolic pathway. **(C)** Relative expression of metabolites related to glycerophospholipid metabolic pathway. **(D)** Relative expression of metabolites related to sphingolipid pathway. #*P* < 0.05; ##*P* < 0.01; ###*P* < 0.001, compared to Model group.

Mongolian Medicine Qiqirigan-8 intervention significantly regulated the expression of metabolites related to glutathione metabolism, butyric acid metabolism, sphingolipid metabolism, and glycerophospholipid metabolism in NAFLD mice. Where MMQ-8 treatment resulted in an overall increase in circulating glutathione (Figure 9A). Fumaric acid and 2-Oxoglutaric acid were significantly increased in butyric acid metabolism (Figure 9B). Glycerophospholipid metabolism (Figure 9C) in MMQ-8 significantly down-regulated LPC levels and up-regulated PC levels after intervention. In addition, sphingolipid metabolism (Figure 9D) significantly increased in adenosine metabolites. Thus, the above results showed that MMQ-8 significantly improved metabolic pathways.

### 3.10 Integrative analysis of the MMQ-8-altered gut microbiota and metabolites

**FIGURE 10:**
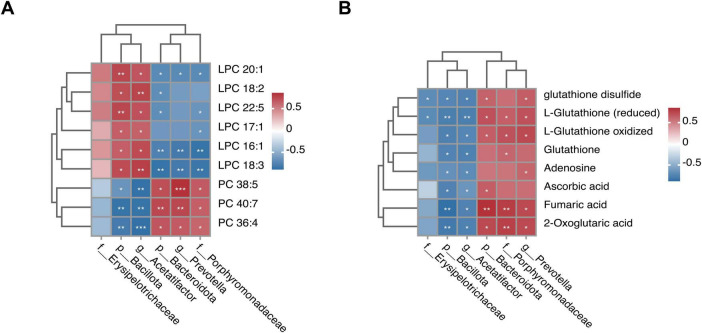
Integrated analysis of Mongolian Medicine Qiqirigan-8 (MMQ-8)-altered gut microbiota and serum metabolites. **(A)** Spearman correlation analysisbetween altered gut microbiota and glycerophospholipid metabolites. **(B)** Spearman correlation analysis between altered gut microbiota and glutathione metabolites, butyric acid metabolites, and sphingolipid metabolites, with red color indicating positive correlation. The blue color indicates a negative correlation. *P < 0.05; **P < 0.01; ***P < 0.001.

To further explore the potential effects of MMQ-8 on the gut microbiota and serum metabolites in NAFLD mice, we performed Spearman correlation analyses to link the altered metabolites to the gut microbiota. Specifically, MMQ-8 up-regulation of *Porphyromonadaceae*, *Prevotella*, and *Bacteroidota* as a whole was negatively correlated with down-regulated LPC levels in the glycerophospholipid metabolism pathway and positively correlated with up-regulated PC levels (Figure 10A). The opposite relationship was observed for Bacillota, Acetatifactor, which was down-regulated after MMQ-8 intervention. In addition, *Porphyromonadaceae*, *Prevotella*, and *Bacteroidota* up-regulated after MMQ-8 intervention were positively correlated with Fumaric acid and 2-oxoglutaric acid, Adenosine, and L-Glutathione. In contrast, *Bacillota* and *Acetatifactor*, which were down-regulated after MMQ-8 intervention, were negatively correlated with all of the above metabolites (Figure 10B).

## 4 Discussion

It is well-known that the high prevalence of NAFLD is becoming a major global health problem (Younossi et al., 2018; Zhou et al., 2020), in which NASH characterized by inflammation and early liver fibrosis leads to cirrhosis, hepatocellular carcinoma (Grander et al., 2023). More importantly, studies have shown that the gut microbiota is involved in the progression of NAFLD (Mouries et al., 2019; Aron-Wisnewsky et al., 2020). Chinese medicine has unique advantages in the treatment of metabolic diseases (Zhang H.-Y. et al., 2021). In Mongolian medicine, which is an important part of Chinese medicine, disorders of the “gut-liver axis” can lead to metabolic diseases such as fatty liver. MMQ-8 is a Mongolian medicine for the treatment of fatty liver disease, and our previous study focused on the effects of MMQ-8 on hepatic steatosis and inflammatory responses in obese rats (Narenmandula et al., 2022). However, the exact effect of MMQ-8 on NAFLD and its mechanism are unclear. In the present study, we firstly detected the *in vivo* active component in MMQ-8 by UHPLC-QE-MS, and found that it has an ameliorative effect on NAFLD. The possible mechanisms are modulation of gut microbiota and serum metabolism.

Serum pharmacochemistry based on UHPLC-QE-MS is a useful analytical tool for revealing the active substances compounded in serum. It can be used for rapid detection of potentially active components in serum after oral treatment with various herbal medicines (Shao et al., 2022). In this study, UHPLC-QE-MS identified 17 potential active serum components of MMQ-8. Furthermore, to clarify the pharmacological effects of MMQ-8 on NAFLD, we utilized a choline-deficient diet to induce mice, leading to oxidative stress and dysregulation of lipid metabolism (Goh et al., 2021). Thereby, rapid accumulation of TG in the mice’s liver and subsequent development of NAFLD. In addition, choline deficiency has been reported to cause hypermetabolism due to sympathetic nervous system outflow to adipose tissue. This leads to increased mitochondrial uncoupling and less efficient energy extraction from nutrients (Xiong et al., 2021). This resulted in elevated liver/body weight in mice, accompanied by increased serum ALT and AST levels that caused liver injury (Lan et al., 2022). In this study, liver TG levels and serum ALT and AST levels were significantly increased in mice fed a choline-deficient diet, resulting in liver injury. In contrast, MMQ-8 significantly reduced TG and serum ALT levels in the livers of mice, which had the effect of reducing hepatic steatosis and protecting against liver injury. Meanwhile, liver pathology analysis showed hepatic steatosis, hepatocyte ballooning, and lobular inflammation in the Model group mice. Liver steatosis and lobular inflammation were significantly reduced by MMQ-8 treatment. Howerver, the MMQ-8 intervention did not reverse the liver/body weight but significantly reduced hepatomegaly. Therefore, MMQ-8 could significantly improve hepatic steatosis and liver injury in NAFLD mice.

It is well-known that the intestinal barrier plays a crucial role in balancing the intestinal flora (Li et al., 2022). NAFLD can lead to disruption of the gut microflora, which in turn produces a variety of toxic metabolites. It can lead to increased intestinal permeability, disrupting the integrity of intestinal epithelial cell junctions and promoting intestinal barrier dysfunction (Wang et al., 2021). We found that inflammatory cell infiltration was visible on the colon surface of mice in the Model group. However, the degree of inflammatory cell infiltration in the colon of mice in the MMQ-8 group was alleviated to different degrees. In addition, under physiological conditions, goblet cells are abundant and have normal secretion function, and the mucus layer is rich in antimicrobial peptides, which can effectively maintain the balance between the gut microbiota and the epithelial cells and prevent infection (Dong et al., 2022). In this study, the epithelial cells and goblet cells in the superficial layer of the colonic mucosa of the Model group mice atrophied and showed different degrees of reduction in number. However, MMQ-8 treatment restored the reduced number of goblet cells compared with the Model group. We evaluated the major intestinal barrier indicators, and it has been reported that the expression of ZO-1, an intestinal barrier indicator, was significantly suppressed in the colon of NAFLD (Zhang et al., 2022). In this study, ZO-1 expression was significantly suppressed in the colon of NAFLD mice and was extensively restored after MMQ-8 treatment. We next examined ZO-1 and Occludin mRNA levels in the colon. We found that consistent with the immunofluorescence results, MMQ-8 significantly up-regulated their expression. Therefore, we can conclude that MMQ-8 can ameliorate intestinal epithelial mucosal barrier disruption in NAFLD mice.

Fecal metagenomics was performed further to clarify the potential mechanisms of MMQ-8 treatment for NAFLD. Our data suggest that MMQ-8 has a significant effect on the gut microbiota of NAFLD mice. The results of principal component analysis (PCA) showed a clear separation of the three groups, with MMQ-8 being closer to the Control group, suggesting that the species composition of the two groups is closer. The elevation of the Chao1 index of the gut microbiota after MMQ-8 intervention was not significant compared to the model group, suggesting that MMQ-8 did not have a significant effect on the species diversity of the gut microbiota. However, the results of β diversity analysis showed that the three groups exhibited different microbiota clustering. This indicates that MMQ-8 did not significantly enhance the α-diversity of gut microbiota in NAFLD mice, but it still altered their β-diversity (Wang et al., 2024). Thus, MMQ-8 treatment-induced changes in gut microbiota structure without affecting the α-diversity of the gut microbiota. Next, LEfSe analysis identified several families that distinguish the gut microbiota groups. Among them, *Erysipelotrichaceae* were significantly dominant in the Model group. *Erysipelotrichaceae* is an important bacterial marker of susceptibility to fatty liver disease caused by choline deficiency (Ren et al., 2021). However, *Erysipelotrichaceae* abundance was significantly decreased after MMQ-8 intervention. Moreover, *Akkermansiaceae* was significantly dominant in the MMQ-8 group. Akkermansia was found to prevent fatty liver and maintain homeostasis in the gut by regulating hepatic lipid synthesis and inflammation, which was positively correlated with physical health status (Kim et al., 2020; Han et al., 2023). In addition, at the phylum level, a significant increase in *Bacillota* and a significant decrease in *Bacteroidota* were observed in NAFLD mice. The *Bacillota/Bacteroidota* ratio (formerly known as the *Firmicutes*: *Bacteroidetes* ratio), an indicator of caloric absorption capacity, was dramatically increased (Zhu et al., 2013). Interestingly, the MMQ-8 intervention significantly reversed the above changes. At the genus level, MMQ-8 can restore gut microbiota dysbiosis by reducing *Acetatifactor* relative abundance in NAFLD mice (Xia et al., 2023). The relative abundance of *prevotella*, a beneficial microorganism negatively correlated with NAFLD severity, was significantly increased after MMQ-8 intervention (Ley, 2016). Similar results were observed at the family level. MMQ-8 treatment significantly increased the relative abundance of *Porphyromonadaceae*, which have been identified as butyric acid-producing bacteria with health benefits (Zhang S. et al., 2021). Thus, the role of MMQ-8 in ameliorating NAFLD may be closely related to its inhibition of harmful bacteria and induction of beneficial bacteria. Further functional analysis of the gut microbiota showed that amino acid metabolism, glutathione metabolism, bile acid metabolism, butyric acid metabolism, sphingolipid metabolism, glycerophospholipid metabolism, fatty acid metabolism, and PPAR signaling pathway were significantly altered compared with the Control group (Rom et al., 2020; Masoodi et al., 2021). At the same time, MMQ-8 treatment was significantly moderated. In addition, there was a high correlation with altered gut microbiota abundance, suggesting that MMQ-8 may alter metabolic pathways by regulating the gut microbiota.

Metabolomics allows access to metabolomic profiles of complex biological systems and the discovery of relevant metabolic pathways for differential metabolites. Thus, it can reveal the pathological process of diseases and has been successfully applied to the study of metabolic diseases (Zhu et al., 2019). In this study, PCA and OPLS-DA showed that different metabolomes were found in the serum of mice treated with MMQ-8 and the Model group of mice. A total of 103 differential metabolites were up-regulated, and 82 differential metabolites were down-regulated in the MMQ-8 group compared to the Model group. We then analyzed these differential metabolites by KEGG enrichment and found significant changes in the pathways of glutathione metabolism, butyric acid metabolism, sphingolipid metabolism, and glycerophospholipid metabolism. Increased oxidative stress in the liver has been reported to be associated with liver injury and NAFLD progression. Increased oxidative stress leads to the depletion of glutathione, the main intracellular antioxidant, which reduces glutathione levels (Muriel, 2009). In this study, circulating glutathione significantly increased and negatively correlated with *Bacillota*, *Acetatifactor*, after MMQ-8 treatment in NAFLD mice. In addition, butyric acid metabolism is involved in the maintenance of intestinal epithelial cells and plays an important role in the regulation of intestinal immune tolerance to antigens (Wang et al., 2020). In this study, butyric acid metabolites significantly increased after MMQ-8 treatment of NASH mice, which was positively correlated with the abundance of butyric acid-producing *Porphyromonadaceae* and negatively correlated with *Bacillota*, *Acetatifactor*. Various studies aimed at exploring hepatic steatosis and changes in the hepatic lipidome in patients with NAFLD have shown that many lipids and lipid species are specifically regulated during the disease stage. There is growing evidence that gut microbiota-derived sphingolipids can regulate hepatic metabolism (Le et al., 2022). One of the sphingolipid metabolites, Adenosine, is involved in hepatic glycolipid metabolic processes (Antonioli et al., 2015). Our results showed that blood Adenosine levels were significantly increased in the MMQ-8 group of mice compared to the Model group and positively correlated with *Prevotella*. PC, the most abundant phospholipid in the liver, has been shown to be reduced in NAFLD patients (Chiappini et al., 2017). Considering that PC is the main phospholipid packaged into very low-density lipoprotein (VLDL), reduced PC levels in NAFLD can inhibit VLDL release and exacerbate hepatic lipid accumulation in NAFLD by stimulating sterol regulatory element binding protein (SREBP) induced re-generation-of-fat. In addition, in the liver, phospholipase A2 (PLA2) converts PC to lysophosphatidylcholine (LPC) by removing a fatty acyl chain. LPC has been associated with a number of deleterious effects in the liver, including increased mitochondrial permeability and decreased mitochondrial fatty acid oxidation. Given the increased hepatic LPC content in NASH patients, this dysregulation may further contribute to the progression of NAFLD (Anari and Montgomery, 2023). However, MMQ-8 significantly increased PC levels and decreased LPC levels in NAFLD mice, thereby significantly modulating glycerophospholipid metabolism. In particular, a high correlation between glutathione metabolism, butyric acid metabolism, sphingolipid metabolism, glycerophospholipid metabolites, and gut microbiota has been identified in both integrated analysis of gut microbiota and non-targeted metabolomics. As mentioned above, MMQ-8 treatment may have beneficial effects in combating NAFLD by modulating the gut microbiota and metabolism.

In conclusion, this integrated microbiota and metabolomics study provides a relationship between MMQ-8 and gut microflora and metabolic dysregulation during choline-deficient diet-induced NAFLD mice. MMQ-8 may be a potential intervention for treating NAFLD by targeting specific microbiota. However, this study aimed to reveal previously unknown roles of MMQ-8 based on gut microbiota and metabolic profiling to provide clues for the prevention and treatment of NAFLD. However, this is a descriptive study of one of the hypotheses proposed by the histological analysis. There are many shortcomings in this study, such as the transplantation of the fecal microbiota of MMQ-8-treated mice to NAFLD mice or germ-free animal Model validation, which needs to be further studied.

## Data Availability

The data presented in the study are deposited in the NCBI repository, accession number PRJNA1201622.

## Footnotes

1
http://www.bioinformatics.com.cn

2
https://www.omicshare.com
